# Editorial behaviors in peer review

**DOI:** 10.1186/s40064-016-2601-y

**Published:** 2016-06-27

**Authors:** Wei Wang, Xiangjie Kong, Jun Zhang, Zhen Chen, Feng Xia, Xianwen Wang

**Affiliations:** School of Software, Dalian University of Technology, Dalian, 116621 China; WISE Lab, Faculty of Humanities and Social Sciences, Dalian University of Technology, Dalian, 116085 China

**Keywords:** Referee, Editor, Agent-based model

## Abstract

Editors play a critical role in the peer review system. How do editorial behaviors affect the performance of peer review? No quantitative model to date allows us to measure the influence of editorial behaviors on different peer review stages such as, manuscript distribution and final decision making. Here, we propose an agent-based model in which the process of peer review is guided mainly by the social interactions among three kinds of agents representing authors, editors and reviewers respectively. We apply this model to analyze a number of editorial behaviors such as decision strategy, number of reviewers and editorial bias on peer review. We find out that peer review outcomes are significantly sensitive to different editorial behaviors. With a small fraction (10 %) of biased editors, the quality of accepted papers declines 11 %, which indicates that effects of editorial biased behavior is worse than that of biased reviewers (7 %). While several peer review models exist, this is the first account for the study of editorial behaviors that is validated on the basis of simulation analysis.

## Background

The peer review system is a cornerstone of scientific research enterprise. The development of science in the last century is an partial endorsement of the value of peer review (Alberts et al. 2008). Unfortunately, peer review systems have recently been under severe strain potentially contributing to cases of misconduct and fraud, such as the stem cell scandal in Science (Crocker and Cooper 2011) or the more recent open-access journals investigation by Plunk (2013). Some of the potential drawbacks result from the fact that the peer review system is a complex social interaction where scientists interact in various roles as journal editors, authors and reviewers in a decentralised, scarcely transparent and relatively unregulated system. Thus, the different behaviours (e.g., positive and negative) of scientists may result in unpredictable collective outcomes in terms of quality and fairness of the reviewing process (Martins 2013). Meanwhile, peer review is a cooperation dilemma that lacks transparency and reputational incentives/sanctions. This creates conditions for self-interest behaviours (Xiao et al. 2014).

The main challenge is how to measure the influence of these various social interactions on peer review and then modify the existing systems in order to minimize the negative and bad effects. Generally, most scientists and journal editors have opinions on how to improve this system, nevertheless it is ambiguous to distinguish which method would be most effective without performing large scale experiments. A large body of research have been done on analyzing the validity and reliability of peer review (Benos et al. 2007; Hojat et al. 2003). The pitfalls, bias, and ethics of peer review had been discussed (Lee et al. 2013; Souder 2011). Meanwhile, to study and optimize peer review, many empirical analysis and statistical approaches have been proposed (Bornmann 2011; Gallo et al. 2014; Petchey et al. 2014). Additionally, examining the quality of various social interactions through empirical analysis from a system level is a tedious and context-dependent task (Edmonds et al. 2011). Some researchers begin to simulate the social process of peer review from a modeling perspective (Squazzoni and Takács 2011), so that approximate measures of the phenomenon can be manipulated.

Recent articles, such as Paolucci and Grimaldo (2014), demonstrated that peer review should be viewed as a complex social interaction problem which requires simulations of social systems to investigate it. Social simulations can help to explore the relevance of social interactions and scientists’ behaviours to better understand how peer review systems work. More importantly, social simulations, especially the agent-based model in this paper, can be used to test various scenarios under specific circumstances. A typical example of applying agent-based modeling to simulate science was Gilbert’s model (Gilbert 1997) which succeeds in designing a specialty structure with ‘areas’ of science displaying growth and decline.

An influential simulation of the social factors of peer review was reported by Thurner and Hanel (2011), where the authors studied the effect of rational reviewers, who might not want to see high quality work better than their own published or promoted, with an agent-based model. They found out that a small fraction of incorrect reviewers is sufficient to drastically lower the quality of the accepted publications. They showed how a simple quality-increasing policy can lead to additional loss in overall scientific quality. The same model was applied in Roebber and Schultz (2011), where the authors focused on funding requests instead of peer review of papers.

In Squazzoni and Gandelli (2012), the authors investigated whether the quality and efficiency of peer review is more influenced by scientists’ behaviour or by the type of scientific community structure (homogeneous vs. heterogeneous). They modeled peer review as a process based on knowledge asymmetries and subject to evaluation bias. They also analyzed the reciprocity behaviour in peer review and found out that reciprocity can have a positive effect on peer review only when reviewers are not driven by self-interest motivation and are inspired by standards of fairness. Based on this work, they further studied the mechanisms of peer review in Squazzoni and Gandelli (2013).

Previous modeling approaches on peer review were mostly designed from author–reviewer (Cabotà et al. 2014) or author–reviewer–conference perspective (Allesina 2012), where they simply overlook the impact of editors’ behaviours. However, there is no doubt that editors play an important role during the whole review process. It is known to all that editors are the bridges between authors and reviewers and so play a gate-keeping role. At the very beginning editors decide how to build the reviewer community and how to assign submitted papers to specific reviewers. After reviewing, the final decisions are also made by editors based on reviewers’ comments. Surprisingly, despite its significance, relatively few attempts have been made to analyze editors’ behaviours on peer review.

Few models introduced above consider the effect of editorial behaviours (Cabotà et al. 2014; Day 2015). In relation to editors, a limited number of studies have evaluated publication performance in editorial decision making based on statistical analysis (Maner 2014; van Lent et al. 2014). The work in Schultz (2010) showed how number of reviewers and different decision-making strategies affect the rejection rate of manuscripts. However, their results were achieved from analyzing data involving 500 manuscripts submitted to Monthly Weather Review without a detailed model. In order to achieve a better understanding of how editors affect peer review system/process, we employ an agent-based model to test/evaluate various editorial behaviors.

## Methods

**Fig. 1:**
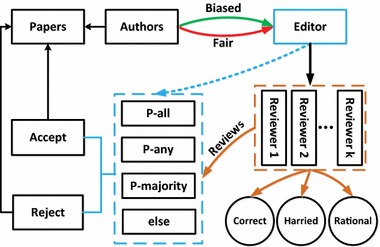
Depiction of the flow of peer review system. Authors write and submit papers to editors. Editors will behave biased if the he has a close relationship with the author. Then editors distribute papers to specific reviewers. Three editorial decision-making strategies are explored: P-all, P-any and P-Majority. Finally, editors will decide whether the submission should be published or not

To explore the influence of editors in peer review, we implement a simulation framework inspired by Thurner and Hanel (2011) in which various editors’ strategies can be tested. Our model is based on a population of agents playing three roles, i.e., editors, reviewers and authors. The author writes and submits paper to editors of a conference/journal. Then the editors allocate submissions to a set number of reviewers chosen from similar-interest authors. The reviewers will evaluate the submission and give a binary decision (‘accept’ or ‘reject’) to editors. Finally, the editors will decide to accept/reject this submission according to reviewers comments and the requirements of the conference/journal. The whole process of peer review can be seen from Fig. 1.

In terms of editors’ behaviors, we specifically consider: (1) Biased versus unbiased behavior: Biased behavior means that an editor will help to accept papers submitted by his friends (or members of same group such as co-authors) regardless of quality. Previous studies mostly focus on how to measure the effects of reviewers bias such as the rational behaviour on peer review, where editor’s bias and honesty are overlooked. (2) Number of Reviewers: Our aim was to test the impact of the number of reviewers on rejection rate and quality of published papers. With more comments from more reviewers, editors can easily evaluate and make a decision regarding a submitted paper. However, the cost of reviewing one paper also rises at the same time. Therefore, we want to measure the effect of different number of reviewers in our proposed model. (3) Decision Strategies: We explore three decision-making strategies for editors: Publish a paper based on ‘accept’ suggestion from all reviewers (P-all), Publish a paper based on ‘accept’ suggestion from any reviewer (P-any), and Publishing a paper based on ‘accept’ recommendation from majority of reviewers (P-majority). It also explores interactions effects between these strategies with different behaviors of reviewers. A set of rules govern the behaviors of these agents to simulate the peer review process (e.g., rational behaviors of reviewers).

### Peer review entities

Our proposed model simulates peer review as a quality selection process where authors, reviewers and editors interact with each other. The authors write papers, submit them to editors for review. Our definition for the scientific quality of authors follows a Gaussian distribution. This means that the scientific quality of authors follows normal distribution at the very beginning, i.e. each author *i* is assigned a $Q^{author}_{i} \in N(Q_{initial}, \sigma ^{2}_{author})$, where $\sigma _{author}$ is the standard deviation of scientific quality of authors and $Q_{initial}$ is the initial average quality of all authors. We consider that in relation to papers published, the scientific quality of authors may increase based on previous published papers by $\gamma$.

The paper entity is the basic unit of evaluation. The quality of submissions follows a normal distribution centred around author quality $Q^{author}$. The quality of a paper submitted by author *i* is $Q^{paper}_{i} \in N(Q^{author}_{i}, \sigma ^{2}_{paper})$, where $\sigma _{paper}$ is the standard deviation of a paper’s quality. The practical meaning of the above analysis is that, with some probabilities good scientists could write average or low-quality papers, and average scientists had some chances to write high-quality papers (Squazzoni and Gandelli 2012).

Each submitted paper is sent to specific independent reviewers, randomly chosen by the editors. Each reviewer belongs to one of the following categories, i.e., the correct, the harried, and the selfish. The correct reviewers will fairly accept high-quality papers and reject low-quality papers. They will recommend publication only for high-quality papers, defined as those which exceed a minimum threshold $Q_{ave}$. This threshold can be calculated in various ways. In this paper, we adopt a simple moving average to compute the threshold shown in Eq. (1) below:

1 $$Q_{ave}(t)=\lambda Q_{ave}(t-1)+(1-\lambda )\overline{Q^{accpt}(t-1)}$$

where $\overline{Q^{accpt}(t-1)}$ indicates the average of all accepted papers in last round. In other words reviewers will accept papers with quality higher than this threshold. The harried reviewers behave like the correct reviewer, but they cannot assess paper quality correctly. They may overrate or underrate a submission. To be specific, their evaluation scores take a standard deviation around the real quality of the paper, i.e. if $N(Q_{j}^{submit}, \sigma ^{2}_{harried}) \ge Q_{ave}$, where $\sigma _{harried}$ is the harried standard deviation, the harried reviewers will make positive comments. The selfish reviewers are not willing to accept papers better than their own because they know that any scientific research work better than their may weaken their academic reputation (Thurner and Hanel 2011). At the same time they will not accept very low-quality papers. That is, a selfish reviewer *i* will recommend declining a proposal that is either superior to his/her own work or below a minimum quality, i.e. $Q^{submit}_{j} \in [Q_{min}, Q^{author}_{i}]$. From an individual point of view the selfish editor may be a rational one.

The editor, who matches authors and reviewers, might be an importance source of bias and influence in the peer review system. In order to simplify the discussion in this paper we assume that every submission is submitted to one editor. There may be situations in which the editor and authors share common friendship networks, where members belong the same group, such as a co-authorship network (Thurner and Hanel 2011). In the case of analyzing the editorial biased behavior regarding acceptance and quality of peer review, we assume the editor will be biased or fair with probability of $e_{b}$ and $1 - e_{b}$ respectively, where $e_{b}$ indicates that the editor share common friendship networks with $e_{b}$ fractions of authors. The fair editor will allocate submissions to reviewers randomly. The biased editor will treat submissions differently. In such cases, a biased editor will allocate submissions to reviewers within their friendship networks. Consequently, due to editor bias, such reviewers will accept papers more easily. More specifically, the reviewer will reduce the reviewing standard. For example, in the case of an reviewer who is connected with the editor, if $Q^{submit}_{j} \ge \xi \times Q_{ave}$, where $\xi \in [0,1]$, the paper will be accepted. Otherwise, a biased editor will behave similarly to a fair one.

### Peer review process

A population of *N* = 100 productive scientists was used for this simulation, each of which follows one of two roles: author or reviewer. When selected as authors, scientists will write and submit manuscripts while selected reviewers will give comments on submitted manuscripts. The whole peer review process is shown in Fig. 1. Each scientist produces one paper at every time step. The quality of a submitted paper is described above. At every time step, each submitted paper is sent to *K* independent reviewers, randomly chosen from the *N* scientists by the editor. Authors cannot review their own paper. Each reviewer will give a binary recommendation within the same time step: ‘accept’ or ‘reject’ based on which kind of reviewer he/she is. The fractions of three kinds of reviewer are $f_{c}$, $f_{r}$ and $f_{h}$ respectively, with $f_{c} + f_{r} + f_{h} = 1$. Based on these recommendations, the editor will decide whether the submission should be published or not. We explore three decision-making strategies for editors in this paper: P-all, P-any and P-majority.

**Table 1:** Simulation parameters

Parameter	N	T	$Q_{initial}$	$\gamma$	$\sigma _{author}$	$\sigma _{paper}$	$\sigma _{harried}$	K	$\xi$
Value	1000	500	100	0.05	10	5	5	[1, 2, 3, 4, 5]	0.9

All simulation parameters are shown in Table 1. The average quality of researchers is set at the beginning of the simulation at 100. At the first round, half of papers are published. Subsequently, papers will get published following previous rules. After each round, the quality of author will raise by $\gamma$ so that their qualities grow accordingly. This means that scholars will learn from previous work and write higher quality papers next time. Meanwhile, the average quality of submitted papers will rise according, i.e, from 100 to 125 after 300 rounds.

We designed various simulation scenarios to test the impact of editors’ behaviours on the quality of peer review. We examined how editorial behaviours influence the average quality of all accepted papers. By the average quality of published papers we can verify the performance of quality selection of the peer review process. Another metric we measure is the number of all published papers, from which we can verify the acceptance rate of a journal/conference. Furthermore, we show the number of low-quality papers published (i.e., with an intrinsic value less than the average), as an indicator of occasional failures (Lee et al. 2013). While two previous quantities can be seen as measures of quality and efficiency, this one can be considered as a measure of fairness.

## Results

**Fig. 2:**
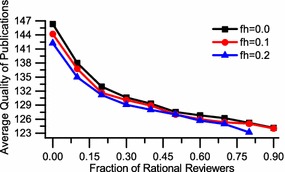
The effect of different fractions of rational reviewers on the average quality of accepted papers. With more fractions of rational reviewers, the quality of publications will drop sharply. Further more, the harried reviewers will slightly bring down the quality of publications

We first examine the effect of rational reviewers in comparison with previous work (Thurner and Hanel 2011). The quality of average accepted papers with different fractions of rational reviewers is shown in Fig. 2 (with all editors being fair). With fractions of over 70 % of rational reviewers the selection mechanism in the reviewing process is almost the same with random selection. Figure 2 further illustrates the situation for two fixed fractions of harried reviewers $f_{h}=0.1$ and 0.2. Obviously, the harried reviewers will slightly bring down the accepted paper quality.

**Fig. 3:**
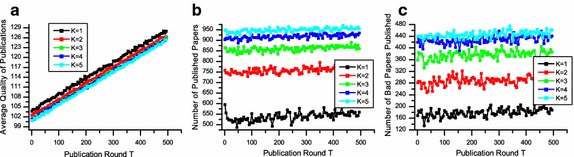
The performance of P-any in terms of average quality of publications (**a**), number of published papers (**b**) and number of low-quality papers published (**c**). The number of reviewers is from 1 to 5

**Fig. 4:**
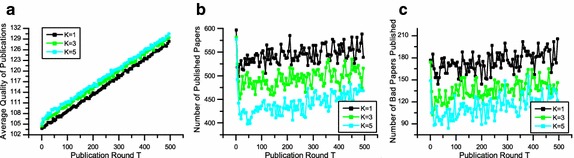
The performance of P-majority in terms of average quality of publications (**a**), number of published papers (**b**) and number of low-quality papers published (**c**). The number of reviewers are 1, 3, and 5 respectively

**Fig. 5:**
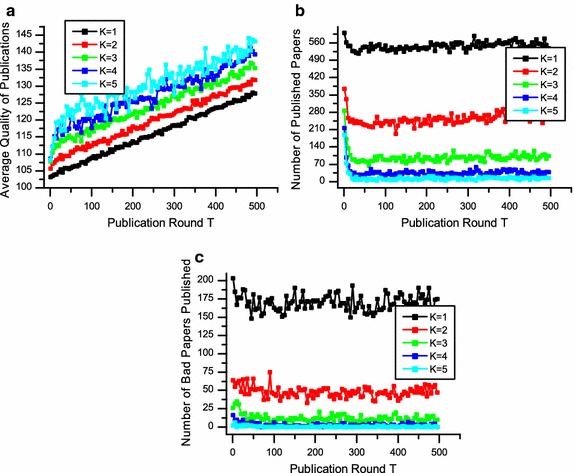
The performance of P-all in terms of average quality of publications (**a**), number of published papers (**b**) and number of low-quality papers published (**c**). The number of reviewers is from 1 to 5

Figures 3a, 4a and 5a depict the average quality of accepted papers. These figures clearly show that differences in editorial strategies matter. Taking *K* = 3 for example, if the editorial decision strategy is P-any, the final average publication quality is 125.882, while the final average publication qualities are 129.348 and 135.455 when the editorial decision strategies are P-majority and P-all, respectively.

In Figs. 3b, 4b and 5b, where we show the number of accepted papers, indicate that with when the average publication quality increases, the number of publication decreases accordingly. The reason is that given the number of reviewers is stable, when editors are looking for more positive reviews, some submissions of relatively low quality will be eventually rejected. Thus the number of accepted papers decreases, while the average quality increases.

To measure the fairness of different strategies, we examine the number of low-quality papers published shown in Figs. 3c, 4c and 5c. Considering *K* = 3 for example, the number of low-quality papers published are 405, 146 and 21 when the strategies are P-any, P-majority and P-all, respectively. We can see that if reviewers seek for strict quality selection by P-all or P-majority, the number of low-quality papers published will decrease. In other words, the reviewing process will be more fair. However, if the decision strategy chosen by an editor is too rigorous, the number of accepted papers may be too small, which may lead to a low acceptance rate.

The above analysis shows that different decision strategies adopted by editors will dramatically affect the performance of peer review in terms of average quality of published papers, number of published papers and low-quality papers published. The results suggest that P-all and P-majority perform better than P-any in terms of quality and fairness.

Figure 3 also depicts the performance of different numbers of reviewer *K* on peer review, where the decision strategy is P-majority. We can see from Fig. 3a that as *K* increases, the average quality of publications upsurges slightly. Meanwhile, both the numbers of published papers and the low-quality papers published decline in accordance. The results indicate that with a higher number of reviewers, the quality and fairness of peer review will be better and more effective. Figures 4 and 5 also show similar trends of various *K* in terms of quality, number of publication and low-quality papers published.

**Fig. 6:**
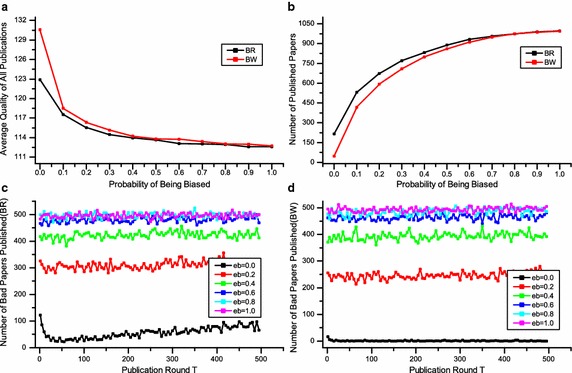
The outcomes of editorial biased behavior, using K = 3 and P-majority. **a**, **b** Depict the average quality of publication and number of published papers under BR (*red line*) and BW (*black line*) respectively. The number of low-quality papers published is shown in **c** BR and **d** BW

Finally, in Fig. 6, we show the results from a simulation of editorial biased behaviour. We set *K* = 3 and the initial decision strategy is P-majority. The simulations have been set into two scenarios that we call bias with rational reviewers (BR) and bias without rational reviewers (BW). These scenarios can help us to better understand the editorial biased behaviour with and without rational reviewers.

Figure 6a shows the average quality of publication with the increasing of $e_{b}$. In both the BR condition (the black line) and BW condition (the red line), the average quality drops accordingly with more fractions of biased editors. It can be seen from Fig. 6a that a small fraction of biased editors (10 %) brings the quality down. To be more specific, from 130.5 to 118.4 (BW) and from 122.9 to 117.6 (BR). The average quality of publications is even below the average quality of all submissions when $e_{b} \ge 0.2$. Figure 6a further indicates that effects of editorial biased behaviour will be worse with rational reviewers.

Figure 6b depicts the number of publications with the increasing of $e_{b}$. Similar to Fig. 6a in both the BR condition (the black line) and BW condition (the red line), the number of publications increases accordingly. When $e_{b} \ge 0.2$, the number of all publications is more than 500, which means that the peer review system does not work at all.

The numbers of low-quality papers published under BR and BW scenarios are shown in Fig. 6c, d, respectively. Considering the BW condition in Fig. 6d, for example, when $e_{b} = 0$, which means there is no biased editor, the number of low-quality papers published is almost zero. While $e_{b} = 0.2$, the number of low-quality papers published increases to around 250. With more biased editors, e.g. $e_{b} =0.4$, almost half of all publications are low-quality papers. The trend under BR condition is similar to that of BW condition, but the performance of peer review under BR condition is worse in comparison to that of BW condition. For example, when the $e_{b} = 0.2$, the number of low-quality papers published is around 300 in BR scenario and 250 in BW scenario. In summary, our results show that the editorial biased behaviour hugely influences the performance of peer review systems.

## Discussion and conclusion

This paper focused on documenting the potential influence of the interaction of social forces between editors and reviewers in the peer review system using an agent-based model. We extended previous study by considering the effect of editorial behaviours on peer review. We examined the effect of editorial behaviours on the average quality of all published papers, the number of published papers and the number of low-quality papers published.

Our proposed model confirmed that even a small fraction of rational reviewers may dramatically distort publication quality (Thurner and Hanel 2011). We also found out that different editorial decision strategies affect the performance of peer review in terms of average quality of published papers, number of published papers and low-quality papers published. The situation slightly changed if editors choose reviewers with different characteristics (the fair, the selfish and the harried) for specific manuscripts. Our results suggest that with more reviewers, the quality and fairness of peer review will be better. However, the cost of the review process also increases at the same time. Especially in recent years, more and more papers are being submitted, some reviewers even decline to review others’ manuscripts, which leads to a tragedy of commons (Hochberg et al. 2009). Consequently, when making a decision on the number of reviewers, editors should be aware that though more reviewers would benefit peer review, the cost should also be taken into consideration (Bianchi 2015). Therefore, the conference/journal should nominate a suitable number of reviewers and make strategic decisions based on their specific requirements. For example, if you need a relatively high acceptance rate you may choose P-majority or P-any.

Furthermore, we investigated the effect of editorial biased behaviors. We found out that peer review outcomes are significantly sensitive to editorial biased behaviour. Even a small fraction (10 %) of biased editors may do harm to the performance of peer review (11.01 %), which is worse than that of biased reviewers (6.81 %). Specifically when $e_{b} \ge 0.2$, the number of all publications is more than 500, which means that the peer review system is not better than random selection. Our results show that the biased behaviour of editors are even more serious than that of rational reviewers. This is because the impact of rational reviewers can be mitigated by increasing the size of reviewer sample (i.e., the number of reviewers per submission). Thus, we suggest that multiple editors could be recommended so that editorial bias can be reduced.

In reality, peer review is a complex social system. In this paper, we only focused on the quality selection aspect of peer review and the interactions between authors, reviewers and editors, which is lack of a sensitivity analysis for the parameters and simulations. In reality, peer review is a complex system and is not a one-step binary accept/reject precess. We ignored other potential benefits such as improvement of reviewing skills, improvement of manuscripts after reviewing and competition among conferences, etc. In addition to considering the above features of peer review systems in future, we also plan to simulate and analyze our model in more reality scenarios, e.g. with real peer review data.
